# What Is the Good of the College?

**Published:** 1920-11-20

**Authors:** 


### THE MATRONS' AND SISTERS' DEPARTMENT

### WHAT IS THE GOOD OF THE COLLEGE?

This is not an untimely question. Lord Knutsford said it was one which people frequently put to him, and though he gave from one point of view an excellent answer to it, there is room for a wider discussion on this elusive remark, so greatly in favour when any new force makes itself apparent.

One of the most curious traits in human nature is the extent to which individuals are capable of hiding their eyes to the benefits they are actually enjoying. It seems sometimes as though no argument, no boon, however rich, will reveal to these folk the sources from which they derive their blessings. Citizens of the British Empire even are to be found wondering what good it is to them to be alive, safe, and free under the arm of the law they affect to despise. And an instance is known to us of one who was so little conscious of the protection she enjoyed as to re-invest her entire property in German securities a couple of years before war broke out. That kind of individual we shall have always with us, the type which hugs a personal grievance, and suspects all that soars beyond their limited intelligence.

The right way of regarding all the varied organisations with which men and women associate themselves for their mutual help and to forward the work of the world is to regard them as a test. "The good of it" is the use each member has the wit to make of it; it is first individual, and then collective. To make no use of the things which should be a source of strength is to stand self-convicted. To see no good in the good things with which life surrounds one is to be blind and deaf and to come short of attainment.

Setting aside for the moment the abstract matters with which the College of Nursing is concerned, individual members cannot escape the responsibility of finding out for themselves what use they can make of the College. What, it may be asked, does one expect to get out of a guinea? Possibly a new hat, or a couple of visits to the theatre, or board and lodging for a day, or a railway journey. But from the guinea spent in securing entry to the College Register the benefits endure for a lifetime. The only condition is that the member shall take the trouble to listen when she is told what they are, and that she should make use of the privileges placed within her reach.

We welcome the institution of a voluntary subscription as a means of inducing many dormant members to reflect upon the opportunities within their reach through the payment of that guinea. The punctual payment of the small annual subscription is no bad test of loyal membership and awakes remembrance. But the true member will not stop short at that. Unless she subscribe to the Bulletin, which will bring her news of College doings, she will be but half-hearted after all. She should not be content to enjoy the abstract advantages alone which accrue to her, whether she will it or not, from her connection with the College. She should cast her eyes round, and consider how best she may widen her life through the social and professional advantages it opens out to her. How many of the College members take the trouble to seek out and join their local centre? How many attend the meetings arranged for their benefit? Right through the whole organisation there is evidence of slackness, of a drawing back from active participation, of, in short, the existence of a "what is the good of it?" spirit. Some eager workers are tempted to reply, "Nothing is any good to *you*, nor ever will be," but, of course, this also is entirely the wrong spirit. Inertness is not to be countered by vehemence. It will only yield to patient, steady educational work.

Need we be surprised at the deadly passive resistance to outside interests observable in the rank and file of nurses? It is one of the main defects of the nursing service. It is the spirit which the College of Nursing exists to combat. The whole trend of most nurse training-schools in the past was in the direction of entire concentration on work. Literature, music, art, science (save as it touched disease) were ruled out. Recreation appealed to tired-out women chiefly through the theatre and the teashop. Family interests were regarded askance. Male friendships were barred. And after years of this intensive existence women were launched on a career which in many of its features was a mere continuance of the training-school routine. Is it to be wondered at that nurses are not yet prepared to taste all the privileges which are flowing in upon them through the medium of the College? It will be a great triumph to rid nurses of their recoil from mental effort, and set each member reflecting what use she can make of the College founded in her interests, what use the College can make of her.

